# Demographics and disparities in cardiomyopathy and ischemic heart disease mortality among U.S. adults >65 years: CDC WONDER 1999–2024 analysis

**DOI:** 10.1097/MS9.0000000000005648

**Published:** 2026-10-27

**Authors:** Yumna Fatima, Muhammad Qasim, Muhammad S. Hanif, Mohammad A. Hassan, Izma K. Ali, Sadia Tameez-Ud-Din, Iman Osman Abufatima, Sohaima Kamal, Taha Alam

**Affiliations:** a Department of Internal Medicine, Shaheed Mohtarma Benazir Bhutto Medical College Lyari, Karachi, Pakistan; b Department of Medicine, Dow University of Health Sciences, Karachi, Pakistan; c Department of Medicine, Gomal Medical College, Dera Ismail Khan (D.I. Khan), Khyber Pakhtunkhwa, Pakistan; d Department of Internal Medicine, Foundation University Medical College, Islamabad, Pakistan; e Department of Medicine, University of Medical Sciences and Technology, Khartoum, Sudan

**Keywords:** cardiomyopathy, ischemic heart disease, mortality trends, older adults, United States

## Abstract

**Background:**

Ischemic heart disease (IHD) and, at its progression, ischemic cardiomyopathy are leading causes of cardiovascular mortality globally. Despite progress in prevention and treatment, large disparities still exist by age, gender, race, and geography, especially among older adults.

**Methods:**

We analyzed deaths related to cardiomyopathy in older adults with IHD in the United States (1999–2024), focusing on demographic and regional differences. Centers for Disease Control and Prevention WONDER Multiple Cause of Death data were used, including individuals aged ≥65 years with IHD listed as a primary or contributing cause of death. Age-adjusted mortality rates (AAMRs) were calculated, and Joinpoint regression was used to calculate annual percent changes (APCs) across demographic and geographic strata.

**Results:**

From 1999 to 2024, AAMRs have fallen from 39.3 to 14.1 per 100 000. A significant drop was recorded between 2002 and 2018 (APC: −4.94%; *P* = 0.004), with a plateau observed from 2018 to 2024. Male mortality was twice as high as that of females (18.7 vs. 10.0 in 2024). NH Black populations had the highest mortality rate in 1999 (42.7), which narrowed by 2024 (17.4); however, disparities continued. Geographically, the South and non-metropolitan areas exhibited the greatest mortality, with slower reductions. One significant shift in trends was in the type of place of death, with a decrease in hospital deaths and an increase in deaths at home and in hospices.

**Conclusion:**

Persistent disparities between sexes, races, geographic regions, and levels of urbanization underscore the need for specific and targeted public health interventions, equal access to care, and refocusing cardiovascular health initiatives on high-risk participants.

**Graphical abstract available at:**

https://links.lww.com/MS9/B434

## Introduction

Ischemic heart disease (IHD) and ischemic cardiomyopathy (ICM) are among the major causes of morbidity and mortality worldwide. Previous reports have estimated mortality ranging from 6600 to 279 000 deaths per country annually in developed countries and 20 000–370 000 deaths per country annually in underdeveloped countries, highlighting the substantial global burden of these conditions^[^1,2^]^. One of the most serious complications of IHD is ICM- a condition characterized by left ventricular dysfunction that develops from a previous history of myocardial infarction or myocardial ischemia. The pathophysiology mainly involves blockage of the coronary arteries, leading to coronary artery disease that interferes with the blood supply to the myocardium, causing ischemia and myocyte death and resulting in ICM. This process reduces cardiac output and contributes to adverse ventricular remodeling, ultimately resulting in heart failure with reduced ejection fraction^[^3^]^. Currently, coronary heart disease and its progression to cardiomyopathy are increasingly prevalent in the younger population (<55 years), although they have traditionally been associated with the elderly and high-risk patients with multiple comorbidities such as diabetes, obesity, and hypertension^[^4^]^. Key risk factors include modifiable conditions such as hypercholesterolemia, hypertension, metabolic syndrome, and non-modifiable factors such as age, gender, and genetics^[^4^]^.

HIGHLIGHTS

- Twenty-five-year national analysis of cardiomyopathy and ischemic heart disease–related mortality using Centers for Disease Control and Prevention WONDER data.
- Age-adjusted mortality declined until 2018, rose during the COVID-19 pandemic, and then modestly decreased.
- Higher mortality was observed among men, older adults, and non-Hispanic Black individuals.
- Significant regional and urban–rural disparities persist across the United States.
- The high prevalence of unspecified cardiomyopathy subtypes indicates gaps in diagnostic reporting.

IHD caused 9.0 million deaths globally in 2021, accounting for 18.6% of total worldwide mortality, as per the World Health Organization report^[^5^]^. However, while declining rates of death in developed countries such as the United States, the United Kingdom, and Australia are attributed to advanced healthcare systems, Eastern European countries and Central Asia have some of the highest IHD prevalence rates globally – and IHD remains a leading cause of death in developed nations^[^6^]^. In 2021, there were 31.1 million incident cases of IHD globally, with 247 million prevalent cases^[^7^]^. The number of deaths caused by cardiovascular diseases (CVDs) has increased significantly, from 12.1 million in 1990 to 20.5 million in 2021^[^8^]^. Recent reports indicate that ICM presents a major burden on the populace, and in 2024, estimates of the prevalence of CVD show that it already affects 48.6% of adults older than 20 years (based on 2020 data reported in 2024), as indicated by the Global Cardiovascular Disease Statistics Report, with the prevalence of CVD increasing with age in both sexes^[^9^]^.

All these disastrous statistics highlight the huge impact of ICM due to its role in the significant increase in healthcare expenditures^[^10^]^. Spending on IHDs – including the healthcare costs of chronic heart failure due to ICM – is estimated to increase by 41%, from $126.2 billion in 2010 to $177.5 billion in 2040^[^10^]^. IHD and cardiomyopathy frequently coexist, particularly in older adults, given that chronic myocardial ischemia is a well-recognized contributor to ventricular dysfunction and heart failure. When present together, these conditions carry a disproportionate burden, where patients tend to experience worse outcomes, greater healthcare utilization, and higher mortality than those with either condition alone. Characterizing mortality trends in this population is therefore important for identifying high-risk groups and guiding targeted public health responses.

This paper will take an extensive approach to reviewing a 24-year period of trends in IHD-related and ICM mortality between 1999 and 2024, with primary interest in regional and geographic differences across the United States among American adults. This approach may help identify populations experiencing disproportionately high mortality rates, which accentuates the importance of specific public health interventions and equal allocation of resources to reduce health disparities, especially among elderly populations with low incomes. The research will also be used to assess the effects of previous and current health policies and cardiovascular health programs on mortality rates and to provide additional information on their effectiveness. Our study has generated statistical information to enhance knowledge of the IHD burden among various U.S. populations, providing a foundation for future research and evidence-based interventions to decrease mortality from CVDs and increase the quality of health outcomes.

This study was conducted and reported in accordance with the TITAN (Transparency in the Reporting of Artificial Intelligence) Guidelines (2025). No artificial intelligence tools were used in the conduct, analysis, or reporting of this study^[^11^]^.

## Methods

### Study setting and population

This study used mortality data from the Centers for Disease Control and Prevention (CDC) WONDER mortality database, a comprehensive repository of mortality (death certificate) data for the 50 U.S. states and the District of Columbia. Deaths were classified using the International Classification of Diseases, Tenth Revision (ICD-10), which distinguishes between the underlying cause of death, defined as the primary cause leading to death, and multiple causes of death (MCD), which include contributing comorbidities. We used MCD Public Use records to determine the burden of ICM, a form of myocardial dysfunction secondary to IHD, in persons aged 65 years and older. Cases were included if IHD was listed either as a primary or a contributing cause of death, as defined by the following ICD-10 codes: I20.0–I20.9, I21.0–I21.9, I22.0–I22.9, I24.1–I24.9, and I25.0–I25.8 for IHD, and I42 for ICM^[^12^]^. Adults aged 65 years and older were included to focus on the group most affected and to minimize confounding factors related to aging. In this way, a detailed evaluation of IHD-related mortality was ensured, reflecting both the primary and secondary importance of IHD in fatal outcomes.

This study was exempt from Institutional Review Board approval because it used publicly available, de-identified data from a government-maintained database, with strict adherence to the STROBE (Strengthening the Reporting of Observational Studies in Epidemiology) guidelines.

### Data abstraction

Mortality figures and their associated populations for the years 1999–2024 were collected from the CDC WONDER database, based on age, gender, race and ethnicity, and geographic location (region, state, and location of death). Our analysis is restricted to adults (those aged ≥65 years), divided into 10-year age groups (65–74, 75–84, 85–94, and ≥95 years). Race/ethnicity was categorized into Hispanic, Non-Hispanic (NH) White, NH Black, NH Asian/Pacific Islander, and NH American Indian/Alaska Native (AI/AN). Geographic trends were identified by two different classification schemes: U.S. Census Bureau regions (Northeast, Midwest, South, and West) and the National Center for Health Statistics Urban–Rural Classification Scheme (metropolitan, non-metropolitan, and rural counties). This multidimensional stratification allowed for robust analysis of patterns of IHD-related mortality in cardiomyopathic patients across key demographic and geographic domains while ensuring consistent classification standards across the study period^[^13^]^.

### Statistical analysis

To analyze nationwide trends in IHD-related mortality from 1999 to 2024 in cardiomyopathic patients, we calculated crude and age-adjusted mortality rates (AAMRs) per 100 000 population, stratified by year, sex, race/ethnicity, state, and urban-rural status, with corresponding 95% CIs. Crude mortality rates were determined by dividing the number of IHD- and ICM-related deaths by the corresponding U.S. population for that year. AAMRs were calculated by standardizing IHD-related deaths to the year 2000 U.S. population^[^14^]^. Trends in AAMRs were analyzed using the Joinpoint Regression Program (Joinpoint V 5.0.2, National Cancer Institute), which determined the annual percent change (APC) with 95% CIs^[^15^]^. This software identifies significant variations in AAMRs over time by fitting log-linear regression models at identified inflection points. APCs were considered increasing or decreasing if the slope describing the change in mortality was significantly different from zero based on two-tailed *t*-testing. Statistical significance was defined as *P* < 0.05^[^16^]^.

## Results

Significant shifts were observed in the place-of-death distribution from 1999 to 2024 among older adults with IHD and cardiomyopathy. In 1999, the largest share of deaths (26.8%) occurred in medical facilities, such as inpatient hospital settings. This proportion steadily declined over the years, reaching 2.9% by 2020. However, a marked increase was observed in 2021, when medical facility deaths surged to 26.4%, likely reflecting the impact of COVID-19-related hospitalizations, with levels remaining elevated through 2024.

Home deaths increased substantially, rising from approximately 4.7% in 1999 to 25.2% in 2021, and remaining at 25.1% by 2024, indicating a shift toward home-based end-of-life care. Deaths occurring in hospice facilities also rose – from just 1.3% in 1999 to 10.5% in 2024 – suggesting an increasing preference for, or accessibility of, palliative care options. Similarly, deaths in nursing homes or long-term care facilities increased from 3.4% in 1999 to 9.7% in 2024 (Supplemental Digital Content Table S2, available at: https://links.lww.com/MS9/B433).

### Annual trends in cardiomyopathy among older adults with ischemic heart disease (1999–2024)

From 1999 to 2024, the AAMR decreased from 39.0 to 13.4 per 100 000, while the age-adjusted death rate declined from 39.3 to 14.1 per 100 000. Despite population growth, total deaths decreased from 13 387 in 1999 to 13 190 in 2024. Joinpoint regression revealed a non-significant decline from 1999 to 2002 (APC: −2.32%; *P* = 0.068), a significant decrease from 2002 to 2018 (APC: −4.94%; 95% CI: −6.52 to −4.72; *P* = 0.004), and a plateau from 2018 to 2024 (APC: −0.83%; *P* = 0.288). A transient increase was noted in 2021–2022, potentially reflecting the indirect impact of the COVID-19 pandemic (Fig. 1) (Supplemental Digital Content Tables S3 and S4, available at: https://links.lww.com/MS9/B433; Supplemental Digital Content Figure S1, available at: https://links.lww.com/MS9/B433).

**Figure 1.**
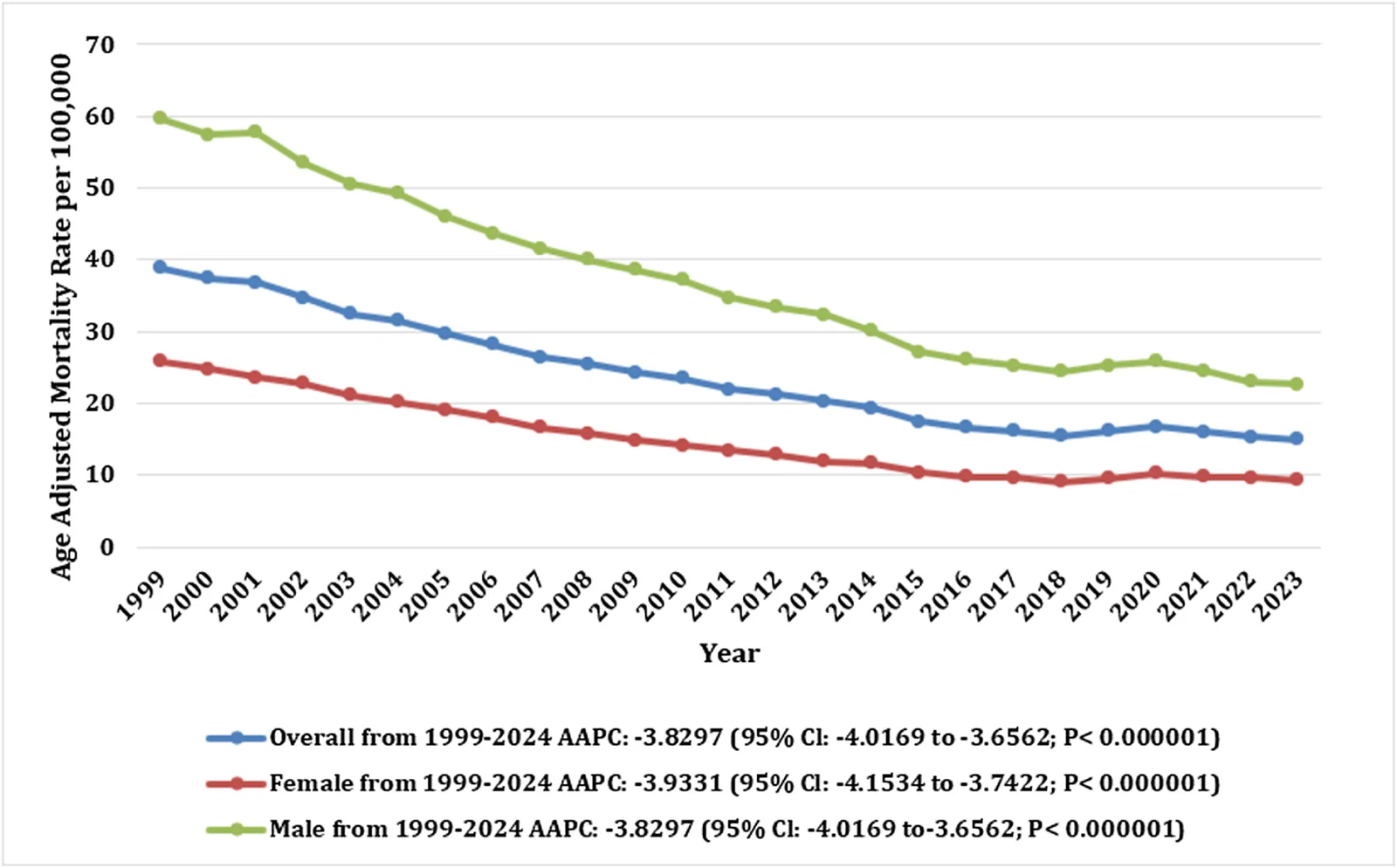
Overall and sex-stratified age-adjusted mortality rates (AAMRs) per 100 000 for cardiomyopathy patients with ischemic heart disease among older adults in the United States, 1999–2024.

### Demographic trends

#### Gender-stratified

The total AAMR reported in 2024 was 10.0 in females and 18.7 in males, with a male-to-female mortality ratio of approximately 1.9:1. Trends showed declining rates in both genders during the study period. Joinpoint regression analysis in the overall population identified three distinct segments: from 1999 to 2002, the decline was not statistically significant (APC, −2.32%; 95% CI, −4.40 to 0.60; *P* = 0.068); from 2002 to 2018, a significant decrease was observed (APC, −4.94%; 95% CI, −6.52 to −4.72; *P* = 0.004); and from 2018 to 2024, the trend plateaued without statistical significance (APC, −0.83%; 95% CI, −2.24 to 2.01; *P* = 0.289). Although the male-female gap narrowed over time, it remained prominent by 2024 (Fig. 1) (Supplemental Digital Content Tables S3 and S4, available at: https://links.lww.com/MS9/B433; Supplemental Digital Content Figure S1, available at: https://links.lww.com/MS9/B433).

#### Race/ethnicity-stratified

The highest age-adjusted mortality in 1999 was recorded in Black or African American populations (42.7 per 100 000), followed by White populations (39.3). Asian or Pacific Islander and AI/AN groups consistently had lower rates. By 2024, rates declined across all groups, with 17.4 per 100 000 in Black and 13.9 per 100 000 in White populations. A significant downward trend was observed among AI/AN populations from 1999 to 2024 (APC: −4.30%; 95% CI: −5.23 to −3.33; *P* < 0.000001). Racial disparities remained evident despite slight narrowing (Fig. 2) (Supplemental Digital Content Tables S3 and S5, available at: https://links.lww.com/MS9/B433; Supplemental Digital Content Figure S2, available at: https://links.lww.com/MS9/B433).

**Figure 2.**
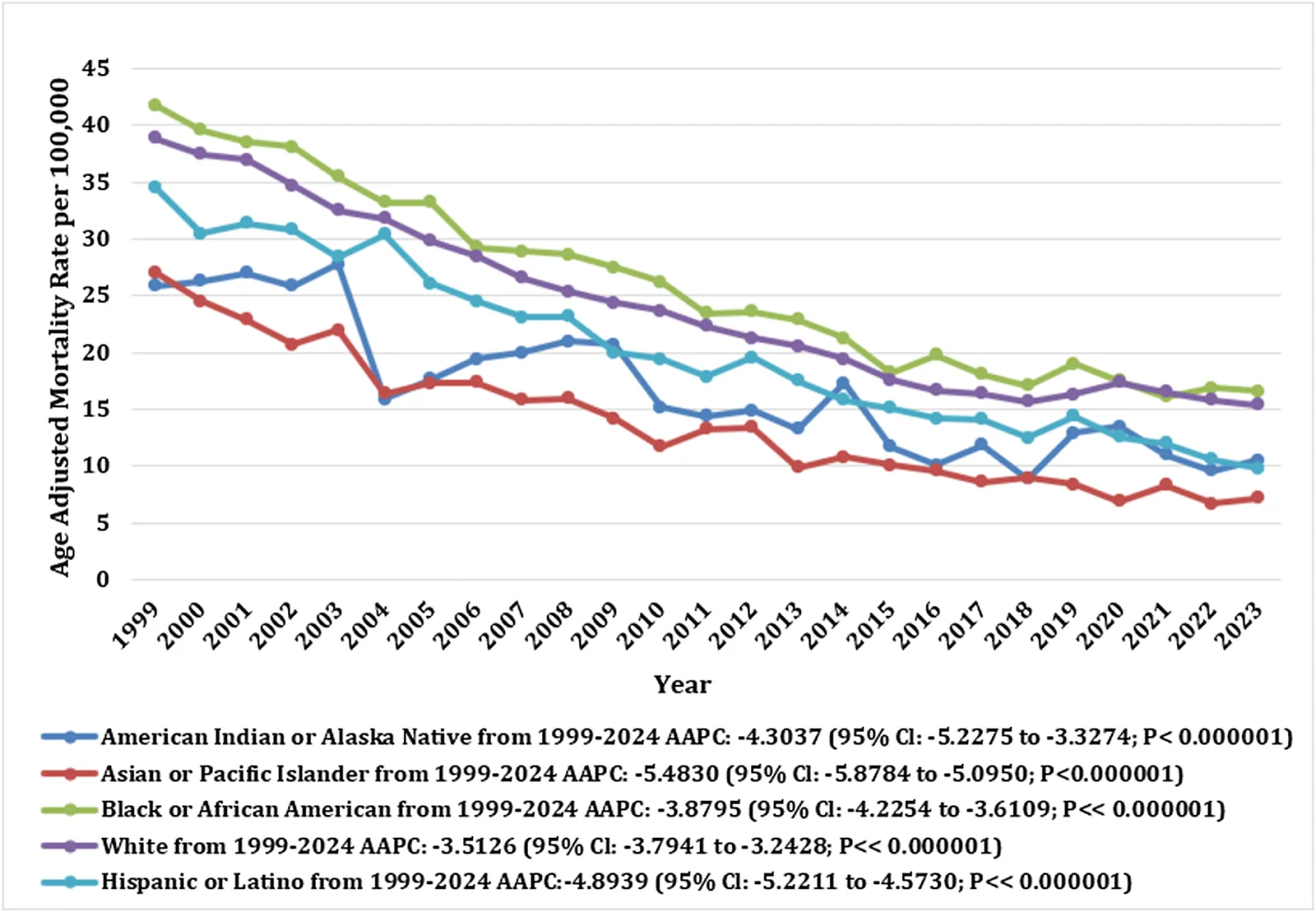
Ischemic heart disease – related age-adjusted mortality rates (AAMRs) per 100 000 among cardiomyopathy patients, stratified by race among older adults in the United States, 1999–2024.

### Geographic trends

#### Regionally stratified

Geographic differences were notable. In 1999, the South recorded the highest AAMR (40.0 per 100 000), while the West had the lowest (36.3). By 2024, the South still led at 14.3, followed by the Midwest, while the West dropped to 12.1. In the Northeast, Joinpoint regression showed a significant decline from 1999 to 2014 (APC: −3.96%; 95% CI: −4.36 to −2.33; *P* = 0.031), followed by a steeper decline from 2014 to 2017 (APC: −7.59%; 95% CI: −9.66 to −4.25; *P* = 0.0032), and a non-significant trend from 2017 to 2024 (APC: −1.90%; *P* = 0.242). While the Northeast and West showed marked reductions, the South and Midwest continued to bear a greater mortality burden (Fig. 3) (Supplemental Digital Content Tables S3 and S6, available at: https://links.lww.com/MS9/B433; Supplemental Digital Content Figure S3, available at: https://links.lww.com/MS9/B433).

**Figure 3.**
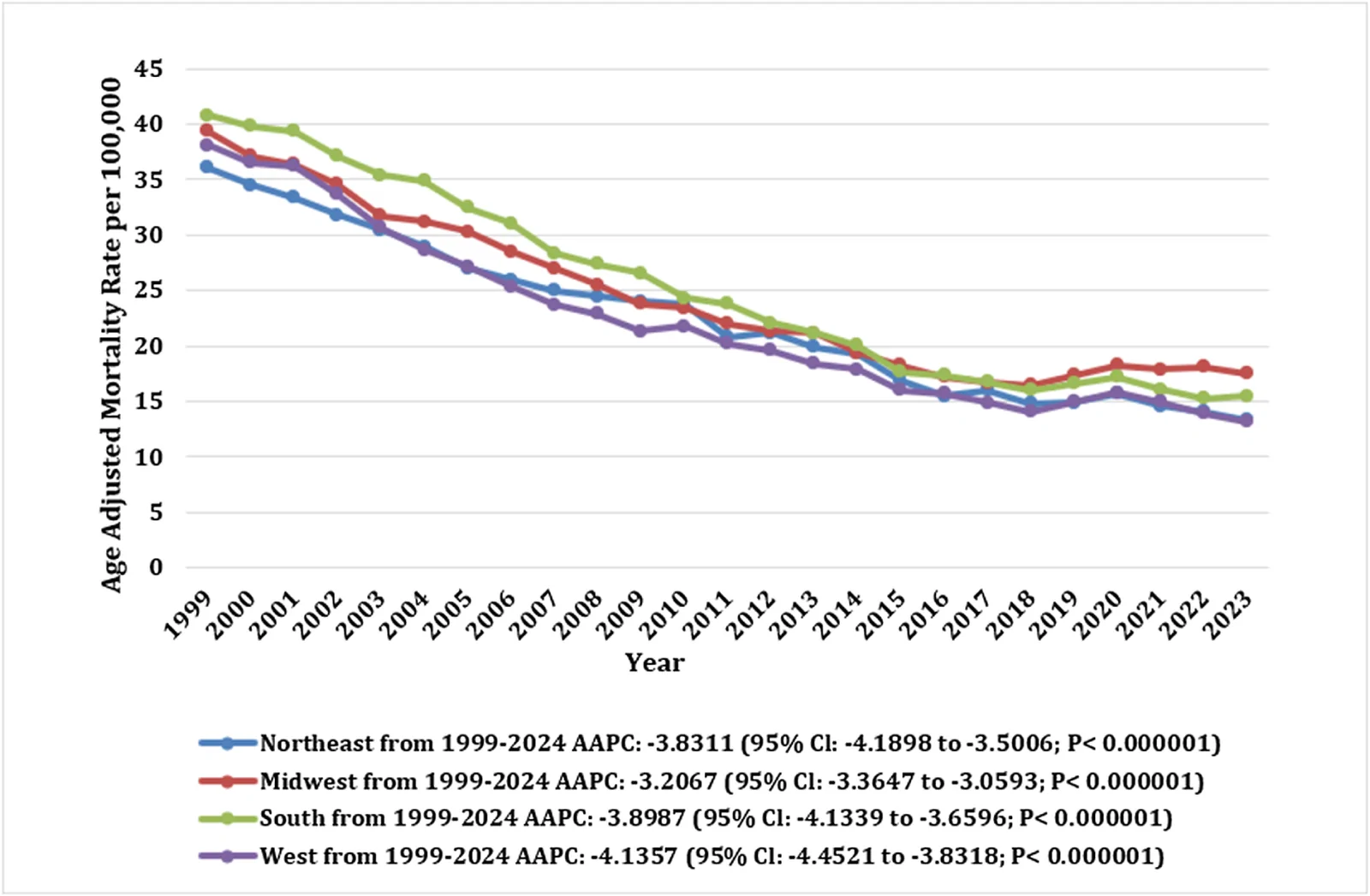
Cardiomyopathy patients with ischemic heart disease – related age-adjusted mortality rates (AAMRs) per 100 000, Stratified by U.S. Census Region Among Older Adults, 1999 to 2024.

#### Urbanization stratified

In 1999, age-adjusted death rates were similar in metropolitan (39.3) and non-metropolitan (36.8) areas. By 2024, the rate in metropolitan areas had declined to 15.8 per 100 000, while the rate in nonmetropolitan areas was slightly higher at 17.9. Among metropolitan areas, a modest decline was observed from 1999 to 2002 (APC: −2.46%; *P* = 0.104), followed by a significant decrease from 2002 to 2018 (APC: −5.04%; 95% CI, −7.43 to −4.88; *P* < 0.000001), and a non-significant trend from 2018 to 2020 (APC: −1.64%; *P* = 0.122). Urbanization trends reflected changes in health care access and disease management (Fig. 4) (Supplemental Digital Content Tables S3 and S7, available at: https://links.lww.com/MS9/B433; Supplemental Digital Content Figure S4, available at: https://links.lww.com/MS9/B433).

**Figure 4.**
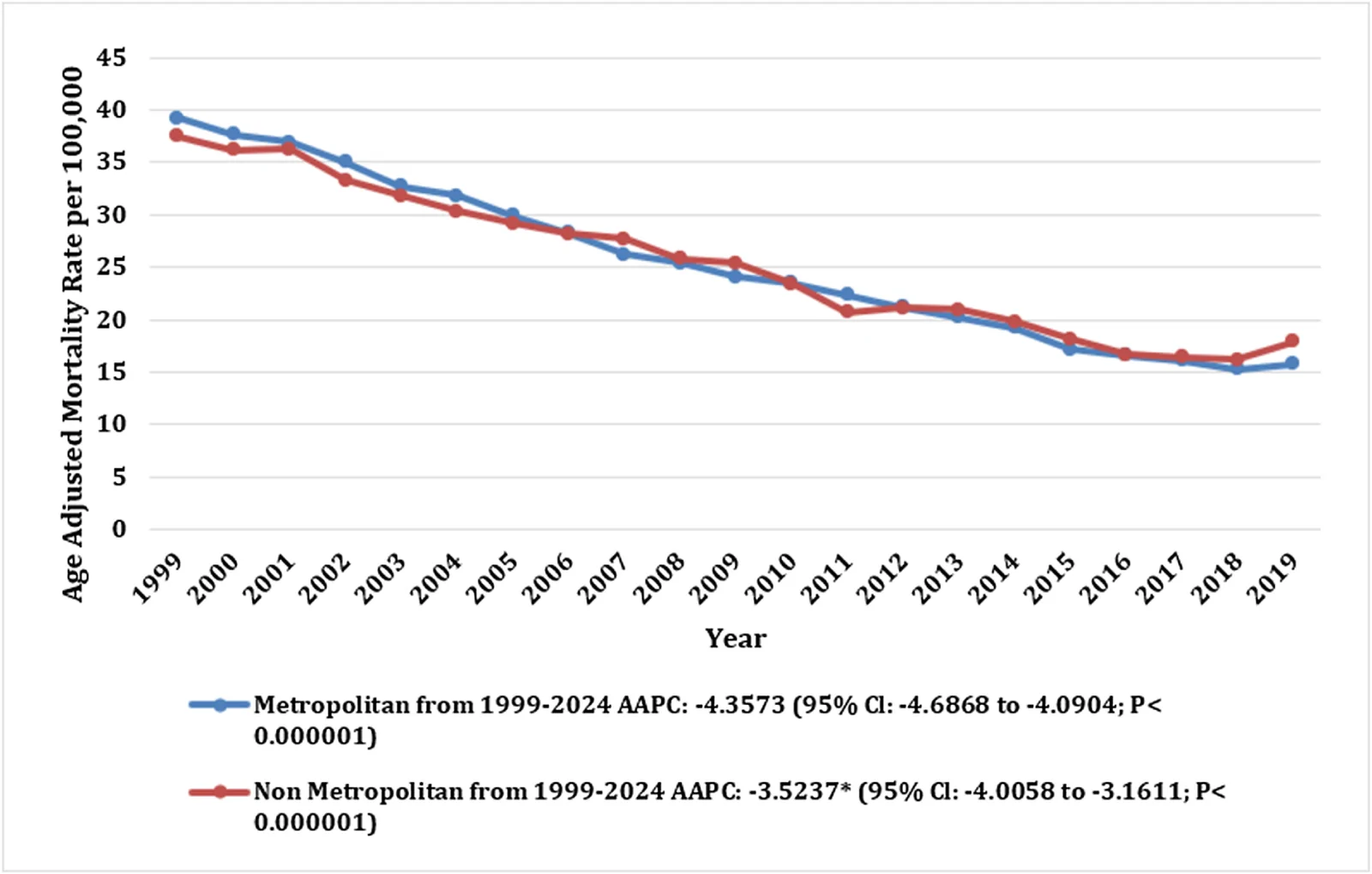
Cardiomyopathy patients with ischemic heart disease – related age-adjusted mortality rates (AAMRs) per 100 000, stratified by urbanization among older adults in the United States, 1999–2020.

### State-level mortality trends

California and Florida reported the highest numbers of total deaths (11.8% and 11.5%, respectively), which correlate with population size. States with the highest AAMR included Florida (35.0), Delaware (34.0), Maryland (33.5), New Jersey (33.4), and Ohio (34.1), while Utah (11.4), Colorado (11.1), and Alaska (15.0) had the lowest. Age-adjusted rates mirrored these trends. Differences likely reflect variations in demographics, healthcare access, and chronic disease prevalence. (Fig. 5) (Supplemental Digital Content Table S8, available at: https://links.lww.com/MS9/B433).

**Figure 5.**
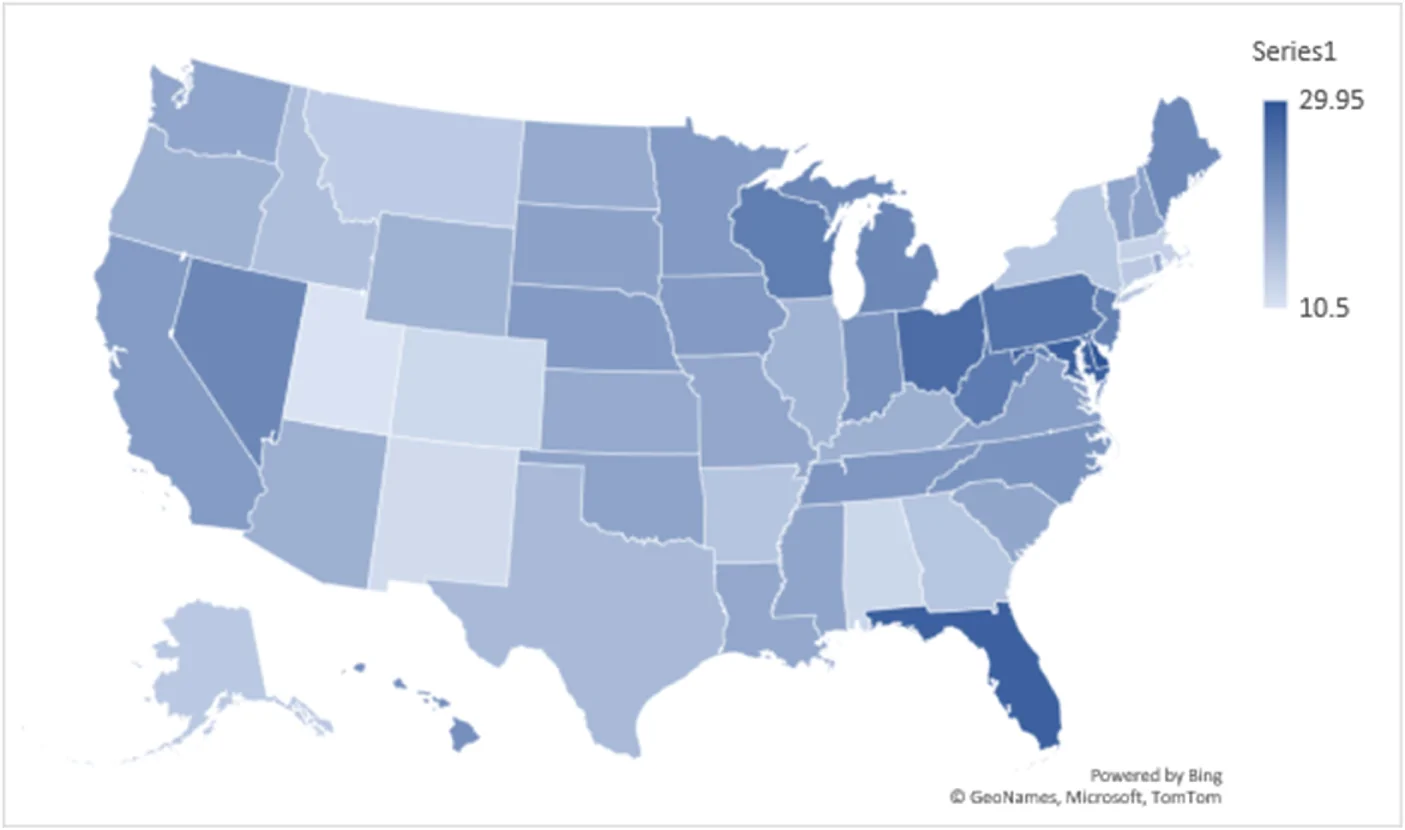
Cardiomyopathy patients with ischemic heart disease-related AAMRs per 100 000, stratified by state among adults in the United States, 1999–2024.

## Discussion

This study assessed long-term patterns of mortality (and place of death) in older people with IHD and cardiomyopathy in the United States over a 25-year period. Broadly, there was a decrease in mortality over much of the study period, followed by a recent stabilization, while there was also a shift in where individuals spent their last days. These findings reflect not only medical advances and the changing delivery of healthcare but also changes in both individual and family approaches to chronic illness and end-of-life care.

The earlier period of declining mortality probably reflects the cumulative effect of improvements in clinical management. Over the past 20 years, standard treatment strategies for IHD and cardiomyopathy have been vastly expanded, with the use of guideline-directed medical therapy, improved cardiac rehabilitation programs, and increased reliance on device therapy where appropriate^[^17,18^]^. The increased focus on secondary prevention, risk assessment practices, and timely revascularization has also contributed to improved outcomes. As a result, patients are living longer with IHD and cardiomyopathy than in past generations (particularly if diagnosed earlier in the disease course).

The following is of note, however, in the slowing, or leveling off, of this decline in more recent years. It implies that, although there has been considerable improvement, it may not be equally distributed across different groups, or that there are barriers limiting long-term gains. This leveling is in line with national trends in cardiovascular mortality, which have begun to level off or even reverse for some populations. Many factors could be contributing: the aging of the population, increasing prevalence of obesity and diabetes, social determinants of health, and limitations in the ability to maintain lifelong adherence to long-term therapy. Despite the availability of excellent treatments, interruptions in continuity of care and follow-up may compromise outcomes^[^19,20^]^.

The short-lived increase in mortality during the COVID-19 pandemic is consistent with the significant strain placed on healthcare systems and chronic disease management during this period, as has been well documented. For many older adults with cardiac disease, routine monitoring, appointments, and medication adjustments were disrupted^[^21^]^. In addition, fear of exposure meant that many individuals delayed seeking care for acute symptoms and presented later and with a more severe clinical status. Notably, COVID-19 itself has been shown to affect cardiac function and exacerbate pre-existing heart conditions^[^22^]^. Taken together, these systemic and biological factors probably contributed to the temporary rise in the number of deaths from cardiomyopathy. The subsequent decline after the first pandemic phase is in line with the stabilization of healthcare services and the re-establishment of outpatient services^[^23^]^.

Gender differences persisted throughout the study period, with higher mortality among men. The trend is not new to cardiovascular studies, and it could be the result of biological susceptibility and access to medical care. Men tend to present with IHD and cardiomyopathy at earlier ages as well as with more severe disease. Lifestyle and behavioral factors, patterns of risk factor accumulation, and past lower rates of engagement in preventive care may also play a role^[^24^]^. Meanwhile, the burden of cardiac disease in older women often develops later, and the course may be equally burdensome following menopause^[^25^]^. Women tend to receive more preventive healthcare, which suggests men’s lower engagement in preventive care is involved in earlier and more severe disease progression^[^26^]^.

Racial variations were also analyzed. A higher rate of mortality was seen among Black older adults, and although rates fell over time, the gap with other groups did not close completely. This pattern probably reflects long-standing inequalities in access to preventive care, continuity of follow-up, and the greater impact of cardiometabolic risk factors experienced at an earlier age^[^27^]^. White older adults also demonstrated a constant decline but kept reporting relatively higher mortality later in the study period (in part because of a higher share of older adults in this group, who were very elderly). Asian/Pacific Islander populations consistently showed lower mortality, which may be attributable to differences in baseline cardiovascular risk, dietary behaviors, and enhanced primary care engagement^[^28^]^. AI/AN groups improved but are still likely to be impacted by limited access to health care in some regions. Overall, the trends identify improvement in all groups, despite their unequal development, influenced by access to health care and social conditions as well as cumulative risk exposure.

Geographic inequalities were also large. The Southern and Midwestern regions consistently had a higher burden of death than the West and Northeast. These regional differences probably indicate differences in healthcare delivery systems, socioeconomic conditions, public health infrastructure, and the burden of comorbid conditions^[^29,30^]^. Lifestyle factors, including diet, physical activity, and the prevalence of smoking, may further affect these patterns^[^31^]^.

Differences between metropolitan and non-metropolitan areas were also evident, with mortality rates higher in non-metropolitan (rural) areas. While the declines in both areas over time were similar, the rate of improvement in rural areas was slower. This may be attributed to the lack of access to health care professionals, particularly specialists, as well as health resources in general^[^32,33^]^. Additionally, rural areas frequently have more difficulty with the implementation of guideline-directed therapies and preventive services^[^34^]^.

Trends in the cause of death also changed significantly over the years. Hospital-based deaths decreased, and deaths in homes, hospice facilities, and long-term care settings increased^[^35^]^. This is likely to reflect both a change in patient and family preferences, as well as wider shifts toward models of palliative and home-based care. However, the spike in hospital-related deaths during the pandemic has highlighted how strains on acute healthcare systems can promote temporary reversals in such trends^[^36^]^.

In contrast, the temporary rise in hospital deaths during the pandemic is probably caused by acute dislocations rather than an about-face in preferences. Hospitals became the portal of entry for acute diseases in response to the novel coronavirus, and outpatient pathways for chronic disease were disrupted. As healthcare systems bounced back, the swing toward home and hospice care settings resumed and supports the interpretation that the long-term trend is one of movement away from hospital-centered end-of-life care.

### Public health implications

These conclusions demonstrate why it is important to invest in preventive cardiology, chronic disease care, and accessible healthcare over the long term. The persistence of demographic and geographic inequalities indicates that specific actions should be implemented, especially in areas with greater cardiovascular risk historically ^[^37^]^. Strengthening outpatient care, medication adherence assistance, and access to cardiac rehabilitation (and ensuring that these services are equitably distributed) will be essential.

In addition, the growing trend toward home- and hospice-based end-of-life care underscores the importance of supporting palliative care infrastructure. Policies that increase the availability of home health services, caregiver support, and community-based palliative programs can help align care delivery with patient preferences and reduce unnecessary hospitalizations near the end of life.

### Study limitations

This study is based on mortality records, which may be subject to variation in the reporting of deaths on death certificates and in the classification of causes of death. Administrative data are unable to capture aspects of disease severity, treatment adherence, social determinants, or comorbidities that can affect outcomes. The epidemiological trends described here should therefore be seen as those of the population rather than the trajectory of individual diseases.

## Conclusion

In summary, over the past two decades, mortality in older adults with IHD and cardiomyopathy has decreased greatly but has recently plateaued, with persistent disparities among demographic and geographic groups. At the same time, the patterns of end-of-life care have shifted toward home, hospice, and long-term care settings, reflecting the changing preferences of patients and patterns of healthcare. Efforts should be maintained to reduce cardiovascular risk, enhance the control of chronic diseases, and provide palliative care to contribute to quality-of-life and equal health outcomes in this aging population.

## Data Availability

The datasets generated and/or analyzed during the current study are available from the corresponding author on reasonable request.

## Study participants

The research is based on de-identified human death certificate data from the CDC WONDER database https://wonder.cdc.gov/mcd.html.
